# Bone Marrow Mesenchymal Stem Cells Expressing Baculovirus-Engineered Bone Morphogenetic Protein-7 Enhance Rabbit Posterolateral Fusion

**DOI:** 10.3390/ijms17071073

**Published:** 2016-07-05

**Authors:** Jen-Chung Liao

**Affiliations:** Department of Orthopedic Surgery, Bone and Joint Research Center, Chang Gung Memorial Hospital, Chang Gung University, Taoyuan 333, Taiwan; jcl1265@adm.cgmh.org.tw; Tel.: +886-3-328-1200 (ext. 2423); Fax: +886-3-327-8113

**Keywords:** bone marrow stem cell, bone morphogenetic protein 7, baculovirus, spinal fusion, rabbit

## Abstract

Previous studies have suggested that bone marrow-derived mesenchymal stem cells (BMDMSCs) genetically modified with baculoviral bone morphogenetic protein-2 (Bac-BMP-2) vectors could achieve successful fusion in a femur defect model or in a spinal fusion model. In this study, BMDMSCs expressing BMP-7 (Bac-BMP-7-BMDMSCs) were generated. We hypothesized that Bac-BMP-7-BMDMSCs could secrete more BMP-7 than untransduced BMDMSCs in vitro and achieve spinal posterolateral fusion in a rabbit model. Eighteen rabbits underwent posterolateral fusion at L4-5. Group I (*n* = 6) was implanted with collagen-β-tricalcium phosphate (TCP)-hydroxyapatite (HA), Group II (*n* = 6) was implanted with collagen-β-TCP-HA plus BMDMSCs, and Group III (*n* = 6) was implanted with collagen-β-TCP-HA plus Bac-BMP-7-BMDMSCs. In vitro production of BMP-7 was quantified with an enzyme-linked immunosorbent assay (ELISA). Spinal fusion was examined using computed tomography (CT), manual palpation, and histological analysis. ELISA demonstrated that Bac-BMP-7-BMDMSCs produced four-fold to five-fold more BMP-7 than did BMDMSCs. In the CT results, 6 fused segments were observed in Group I (50%, 6/12), 8 in Group II (67%, 8/12), and 12 in Group III (100%, 12/12). The fusion rate, determined by manual palpation, was 0% (0/6) in Group I, 0% (0/6) in Group II, and 83% (5/6) in Group III. Histology showed that Group III had more new bone and matured marrow formation. In conclusion, BMDMSCs genetically transduced with the Bac-BMP-7 vector could express more BMP-7 than untransduced BMDMSCs. These Bac-BMP-7-BMDMSCs on collagen-β-TCP-HA scaffolds were able to induce successful spinal fusion in rabbits.

## 1. Introduction

For an unstable spine, successful spinal fusion remains the gold standard among all surgical methods. Autografts and allografts have been demonstrated effectively in spinal fusion surgery, but each carries its limitations. Autogenous bone grafts are limited by the amount of bone available and by significant donor site morbidity [1,2], whereas allogenic bone grafts have a limited osteoinductive property with higher pseudoarthrosis rate in multiple-level spine procedures and carry the risk of disease transmission [3,4]. Bone morphogenetic proteins (BMP) can be extracted from a demineralized bone matrix and used as bone graft substitutes in certain orthopedic surgeries [5]. Nowadays, recombinant human morphgenetic proteins (rhBMPs) are acquired by the cloning and molecular techniques; the United States Food and Drug Administration (FDA) only approved rhBMP-7, and rhBMP-2 can be obtained commercially and applied in certain spinal procedures. RhBMP-2 is indicated for anterior lumbar interbody fusion, and rhBMP-7 is suggested for lumbar posterolateral fusion [6]. A single exposure to a large dose of exogenous growth factor is often required to achieve adequate bone fusion. In the clinical setting, a large dose of rhBMP-7 (7 mg) is required to achieve a one-level intertransverse fusion bilaterally, which implies that the cost is very high for clinical use. Furthermore, local adverse effects such as severe inflammation reactions and unwanted ectopic bone formation have been reported in association with the doses currently used [7,8].

The goal of gene therapy is to convert the target cells into biologically active cells that can produce protein on a continuous basis [9]. Therefore, ex vivo regional gene therapy may be a more efficient tool to deliver a specific protein. Theoretically, mesenchymal stem cells expressing BMP-7 may have potential advantages over the use of recombinant BMP-7 because of (1) the continuous production and delivery of BMP-7 directly to the fusion site for a sustained period of time; and (2) the use of mesenchymal stem cells that have an inherent osteoinductive ability. Bone marrow is a well-established source of mesenchymal stem cells (MSCs) that can differentiate into osteoblasts to stimulate bone healing [10]. Furthermore, bone marrow-derived mesenchymal stem cells (BMDMSCs) have been widely accepted as a cell-base to deliver BMP for enhancing bone healing [11]. In 1995, a baculovirus was first reported in the successful transfection of human hepatocytes [12]. Since then, numerous mammalian cells including neural cells, fibroblasts, chondrocytes, pancreatic islet cells, and human bone marrow stem cells were used to be transfected by baculovirus vectors [13,14,15,16,17]. Hu et al. demonstrated that baculovirus inside the mammalian cells did not induce any obvious cytopathic effects [18].

Given the potentials of baculoviral vectors in gene therapy and our interest in spinal fusion, we tried to construct baculoviral vectors encoding the BMP-7 coding sequence then transduced bone marrow-derived stem cells. The purpose of this study is to test the potential of these stem cells, which are genetically modified by baculovirus to express BMP-7. We tested the hypothesis that a treatment combining collagen sponge-β-tricalcium phosphate (TCP)-hydroxyapatite (HA) composite and baculovirus genetically-modified BMDMSCs over-expressing BMP-7 could enhance spinal posterolateral fusion, as compared with BMDMSCs alone.

## 2. Results

All 18 rabbits used in the study tolerated the surgery well, and no rabbit died before harvest. Thus, all animals completed the experimental protocol, and were included in the final analysis.

### 2.1. In Vitro Study: Enzyme-Linked Immunosorbent Assay (ELISA) for Bone Morphogenetic Protein BMP-7 Production

**Figure 1:**
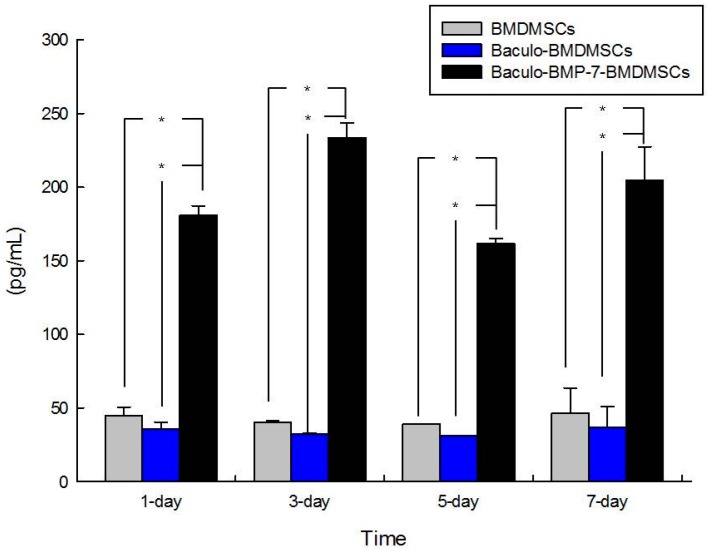
This graph demonstrates the levels of BMP-7 production by bone marrow-derived mesenchymal stem cells (BMDMSCs), Baculo-BMDMSCs, and Baculo-BMP-7-BMDMSCs. Baculo-BMP-7-BMDMSCs secreted significantly more BMP-7 than the other two groups at all the time points. * means a *p* value < 0.05.

ELISA was performed on samples collected at one day, three days, five days, and seven days after transduction. The results showed that baculoviral BMP-7 transduced BMDMSCs secreted mean BMP-7 levels of 187.5 ± 7.8, 242.8 ± 5.5, 165.3 ± 14.7, and 212.4 ± 16.6 pg/mL, respectively, at these four time points. In contrast, BMDMSCs transduced with a baculoviral vector containing a null BMP sequence showed BMP-7 secretion levels of 41.0 ± 9.3, 37.4 ± 8.8, 32.4 ± 6.4, and 39.4 ± 9.1 pg/mL, respectively. BMP-7 production in untransduced BMDMSCs was 47.3 ± 15.0, 45.3 ± 8.1, 44.4 ± 6.9, and 49.5 ± 9.9 pg/mL, respectively, on these four observation days. The baculoviral BMP-7 transduced BMDMSCs exhibited four-fold to five-fold greater BMP-7 production than untransduced BMDMSCs. Compared with BMDMSCs containing the baculoviral vector with a null BMP-7 sequence, BMDMSCs containing the baculo-BMP-7 vector could secrete four-fold to seven-fold BMP-7. Figure 1 illustrates the BMP-7 levels secreted by BMDMSCs, Baculo-BMDMSCs, and Baculo-BMP-7-BMDMSCs at the different time points. Baculo-BMP-7-BMDMSCs produced significantly high levels of BMP-7.

### 2.2. Evidence of Cells Seeded on the Scaffold

**Figure 2:**
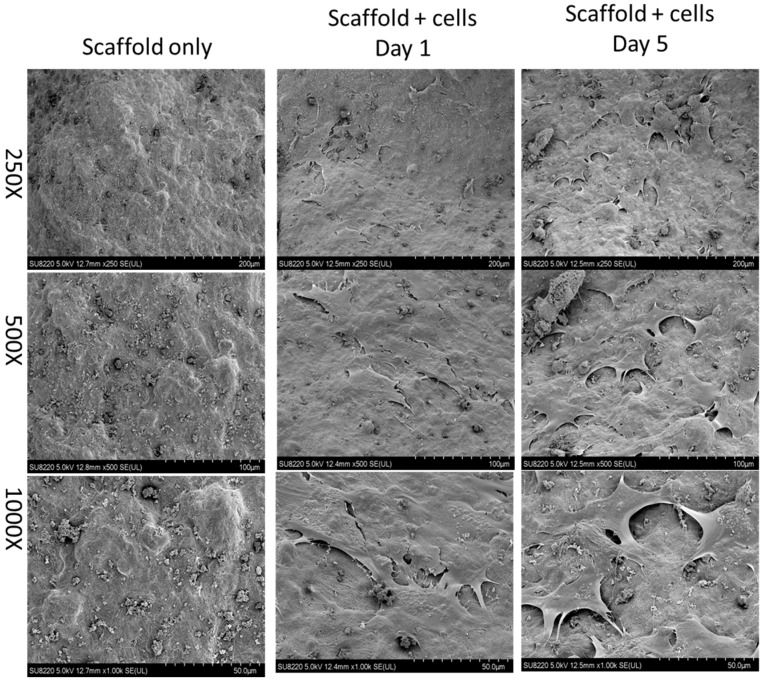
A scanning electron micrograph demonstrates the scaffold without cells and the scaffold with cells (Baculo-BMP-7-BMDMSCs). This image shows that the cells were successfully seeded onto the scaffold.

Before animal studies, test cells including Baculo-BMP-7-BMDMSCs and BMDMSCs were loaded onto scaffolds, and their presence was confirmed by scanning electron micrograph (SEM). Figure 2 illustrates the scaffold alone and the scaffold seeded with Baculo-BMP-7-BMDMSCs under SEM. Even after 5 days of in vitro culture, Baculo-BMP-7-BMDMSCs were still observed on the scaffolds.

### 2.3. Radiographic Evaluation

**Figure 3:**
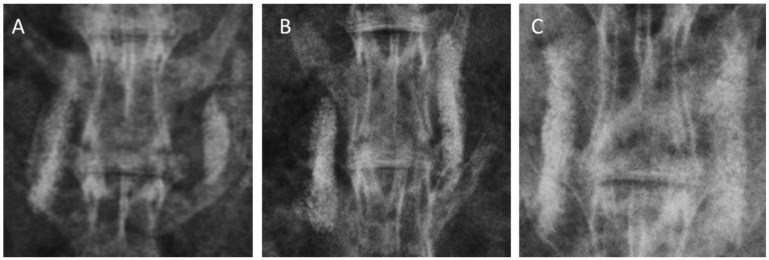
Radiographs of grafted materials in each group after 12 weeks of posterolateral fusion. (**A**) collagen-β-TCP-HA; (**B**) collagen-β-TCP-HA/BMDMSCs; (**C**) collagen-β-TCP-HA/Baculo-BMP-7-B MDMSCs. The residual mineral component of the scaffold was still observed in these three groups. More abundant new bone formation could be observed in the group grafted with collagen-β-TCP-HA/Baculo-BMP-7-BMDMSCs.

**Figure 4:**
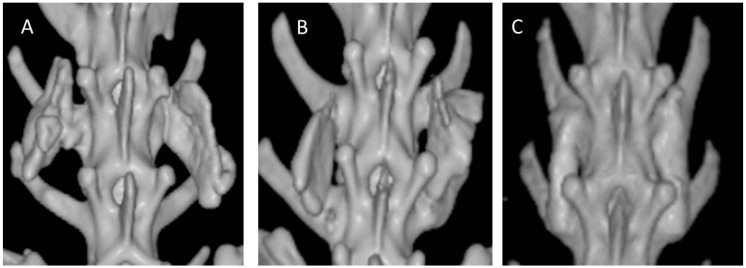
Computed tomography (CT) scans of 12-week specimens from each group. (**A**) collagen-β-TCP-HA; (**B**) collagen-β-TCP-HA/BMDMSCs; (**C**) collagen-β-TCP-HA/Baculo-BMP-7-BMDMSCs. The specimen from the collagen-β-TCP-HA/Baculo-BMP-7-BMDMSCs group had a stronger fusion mass between the inter-transverse process spaces.

Each group included six animals with 12 inter-transverse fusion sites each (for a total of 12 sites), as the right and left inter-transverse spaces were assessed independently. Standard radiography was difficult to demonstrate a successful fusion because the collagen-β-TCP-HA carrier was not completely absorbed and had a strong radio-opacity due to TCP-HA. However, evidence of new bone formation at the margins of the material was observed at 12 weeks in all three groups, and was especially more prominent in Group III. Typical radiographs at 12 weeks following surgery are presented in Figure 3. More accurate assessments of fusion were by determined by computed tomography (CT) scans. Fusion sites with solid calcified materials between the spaces of the transverse process with an uninterrupted bridge were classified as having radiographic union (Figure 4). The radiographic fusion rates determined by CT scans were as follows: Group I 50%, (6/12), Group II 67%, (8/12), and Group III 100% (12/12). Group III significantly showed the greatest fusion rate among all three groups (*p* < 0.001).

### 2.4. Manual Examination

Specimens from Group I revealed non-absorbed collagen-β-TCP-HA attached to the inter-transverse process with fibrous tissue. No obvious bone formation could be seen. Specimens from Group II also showed some non-absorbed carrier inside the inter-transverse process space, but did reveal some bone formation. Specimens from Group III demonstrated more bone formation between the transverse processes. By manual palpation, no animal in Group I was found to have achieved solid fusion (0/6, 0%), which was also true for Group II (0/6, 0%). However, five animals in Group III (5/6, 83%) were found to demonstrate successful unions. Statistical analysis demonstrated a significantly greater spinal fusion rate in rabbits that were treated with collagen-β-TCP-HA/Baculo-BMP-7-BMDMSCs than in those treated with collagen-β-TCP-HA/BMDMSCs or with collagen-β-TCP-HA alone (*p* = 0.012).

### 2.5. Histological Analysis

**Figure 5:**
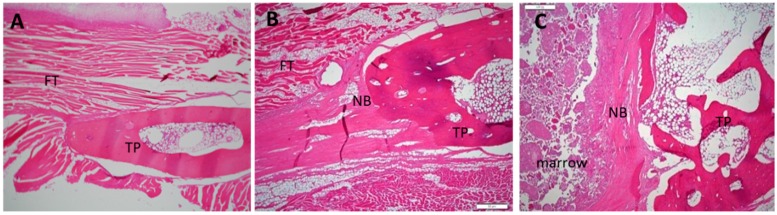
Histological images of 12-week specimens from each group upon hematoxylin and eosin staining. (original magnification: 40×). (**A**) collagen-β-TCP-HA; (**B**) collagen-β-TCP-HA/BMDMSCs; (**C**) collagen-β-TCP-HA/Baculo-BMP-7-BMDMSCs. No lymphocytes or inflammatory cells were observed in these specimens. FT = fibrous tissue; TP = transverse process; NB = new bone.

**Figure 6:**
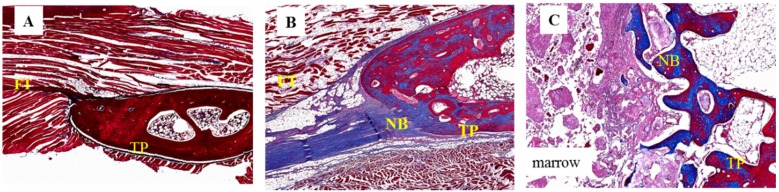
Histological images of 12-week specimens from each group upon Masson’s trichrome staining. (original magnification: 40×). (**A**) collagen-β-TCP-HA; the specimen exhibited thick fibrous tissue at the inter-transverse process space; (**B**) collagen-β-TCP-HA/BMDMSCs; the specimen revealed scattered fibrous tissue with some bone formation and successful fusion; (**C**) collagen-β-TCP-HA/Baculo-BMP-7-BMDMSCs; the specimen showed abundant new bone formation and a successful fused mass at the inter-transverse process space. FT = fibrous tissue; TP = transverse process; NB = new bone.

After manual palpation, all specimens were subjected to a histological study involving hematoxylin and eosin (H & E) staining and Masson’s trichrome staining. No evidence of inflammatory cells or any other adverse reactions was observed in any specimen after H & E staining (Figure 5). By Masson’s trichrome staining, all specimens from Group I demonstrated thick fibrous tissue without new bone between their inter-transverse processes. Successful fusion specimens from Group II demonstrated more bone formation between the transverse processes when compared to specimens of Group I. The fused specimen from Group III showed the most abundant bone formation and scattered bone marrow formation; the specimens were observed to have successfully bridged the L4–5 transverse process (Figure 6).

## 3. Discussion

An ideal bone graft should contain osteoconductive, osteoinductive, and osteogenic abilities [19]. Scaffolds, growths factors, and cells are the three main factors required to create a tissue-engineering construct, which can be used to repair bone defects. The current study demonstrated that the inter-transverse space of the spinal posterolateral area in rabbits could be fused by a tissue-engineered graft containing collagen/β-TCP/HA scaffolds coated with gene-modified bone marrow stem cells. An ideal scaffold for bone regeneration must be biocompatible to allow cell attachment, proliferation, extra-cellular matrix accumulation, and then support bone regeneration in a bony defect or a spinal fusion site [20]. Ceramics, collagen, coralline, and β-TCP are currently used as osteo-conductive graft substitutes [21,22]. The scaffold used in the present study was a composite of collagen, β-TCP, and HA. Collagen is conductive for the deposition of cells and growth factors; β-TCP mimics the trabeculae of cancellous bone, and is developed for vascularization and bone ingrowth; HA is usually coated on artificial implants and increases new bone deposition at the interface. Walsh et al. performed a study to demonstrate the effects of collagen/β-TCP/HA scaffolds on spinal posterolateral fusion in rabbits [23]. Their results showed new bone formation around the implanted material, where bone mineral density and mechanical testing in the group with collagen/β-TCP/HA grafts were higher than those in the autograft group. Even in our study, we found little new bone formation around collagen/β-TCP/HA scaffolds on radiographs in Group I. CT analysis showed that there were six segments in Group I revealing the mineral bridging transverse process. However, these bridging masses were thinner than those in Groups II and III. We believe that the bridging materials were the remaining inorganic materials of the collagen/β-TCP/HA grafts, and not real fused bone, because solid bone masses were not palpable in the harvested specimens of either Group I or Group II. The CT machine that was used in the current study could only provide three-dimensional images for assessing the fusion status, but could not provide fusion mass volume or bone mineral density data for further comparison, and this was one limitation of the present study. In the future, a more advanced machine should be available for image analysis.

Mesenchymal stem cells isolated from bone marrow aspirates provide an unlimited cell source with high proliferation activity and the potential to differentiate into osteogenic cell lineages, which have the ability to generate bone when cultured with dexamethasone, ascorbic acid, and β-glycerophosphate [24,25]. Several studies have shown that culturing BMDMSCs with growth factors could enhance their osteogenic potency [26,27,28]. Mesenchymal stem cells from the bone marrow have been shown to achieve successful spinal posterolateral fusion for more than 10 years. In 2001, Cui et al. demonstrated that cloned osteoprogenitor cells from the bone marrow produced a quicker and larger amount of osseous tissue than mixed bone marrow stromal cells [29]. In contrast, a study from Nakajima et al. showed that bone marrow mesenchymal stem cells without osteogenic differentiation produced weaker posterolateral fusion in rabbits [30]. In our study, BMDMSCs in Group II were not co-cultured with growth factors or any osteogenic solution. Although new bone formation was observed upon histological examination in this group, the osteogenic abilities of BMDMSCs were not strong enough to produce a mature osseous tissue successful fusion.

Genetically modified BMDMSCs that produce growth factors have been shown to successfully induce bone formation in vivo for over one decade [11]. Adenoviral, retroviral, or lentiviral vectors are usually used for gene transfer; and the *BMP-2* gene has been the most popular for study. Hidaka et al. first reported the efficacy of adeno-BMP-7 expressing bone marrow stem cells on spinal fusion in a rat model [31]. The authors found that adenovirus BMP-7 modified marrow stem cells significantly enhanced allograft spinal fusion. Zhu et al. reported that the combined gene transfer of *BMP-7* and *BMP-2* using bone marrow stem cells in a rat model was more effective in inducing osteoblastic differentiation and showed more spinal fusion than individual *BMP* gene transfer [32]. Although adenoviral or lentiviral vectors are popular, they have several drawbacks and safety concerns [33]. The most undesirable effect of the adenoviral or lentiviral vectors was uncontrolled gene expression [34]. In the current study, we used baculovirus as the viral vector because of its efficacy in transducing a broad range of mammalian cells, and the transduced stem cells remained capable of differentiation into various lineages [35]. Furthermore, baculovirus does not replicate in mammalian cells and degrades with time, thus mediating transient gene expression only [36]. In the present study, the baculo-BMP-7-BMDMSCs could secrete more BMP-7 than BMDMSCs in the vitro study. This implied that baculo-BMP-7-BMDMSCs could overexpress BMP-7. From our in vivo observations, neither gross inspection nor radiographic findings showed any undesirable heterotopic bone formation in the spinal canal and other non-surgical segments, which indicates that the characteristics of baculovirus might circumvent the safety concerns associated with other viral vectors. Previous studies have shown that mesenchymal stem cells with baculoviral-mediated BMP-2 delivery could successfully induce healing in a long bone defect model or a in a model of spine posterolateral fusion [37,38]. Similarly, in our study, the transduced BMDMSCs could secrete BMP-7 to stimulate bone repair directly; the secreted BMP-7 might induce these BMDMSCs to transform into osteoblasts and augment the bone healing process. The collagen/β-TCP/HA graft provided an osteoconductive scaffold for the attachment of these gene modified BMDMSCs, prolonged the osteogenic capacity of the cells, resulting in successful fusion. The successful fusion was confirmed by radiographic examination, manual palpations, and histological analyses.

The ultimate goal of gene therapy development is that it can be applied successfully to clinical diseases. As adeno-associated virus (AAV) vectors have been studied for over three decades, clinical trials using AAV vectors have been conducted in cardiovascular disease, ocular disease, and hemophilia [39,40,41]. However, the host immune reaction to AAV vectors and mutagenesis remained a major concern [42,43]. Baculoviral vectors are non-integrating vectors, which limits the risk of insertion mutagenesis [44]. The inability to integrate makes baculoviral vectors well suited to gene therapy applications, where transient transgene expression is preferred, as in cancer or glioma gene therapy [45,46]. Although baculoviral vectors also demonstrate safety and efficiency in engineering human stem cells in vitro or in vivo; however, there is still no clinical trial or application using baculoviral vectors.

Indeed, this study still has limitations. First, the fusion rate as evaluated by radiographs and 3-D CT in the current study only provided qualitative assessment but did not allow estimation of fusion bone volume or bone mineral density for comparison between different groups. In the future, more advanced machines should be used for image analysis. Secondly, as the hosts of these baculoviral vectors were bone marrow stem cells, this study did not detect the viability of these bone marrow stem cells after transduction with baculoviral vectors. Although the current study provided information about BMDMSCs attached to scaffolds for up to 5 days in vitro and BMP-7 production increases in vitro in transduced BMDMSCs, these evidences only demonstrate that transduced BMDMSCs could produce BMP-7, but did not reflect the effects of baculoviral vectors on BMDMSCs viability. Some tests should be included to detect BMDMSCs viability in vitro after baculoviral vector transduction and to observe the long-term viability of the transduced BMDMSCs inside animals. Thirdly, introduction of baculoviral vectors into a host might induce an immune response or an allergy effect [47]. In the present study, baculoviral vectors transduced BMDMSCs in rabbits did not induce an obvious immune reaction as was evidenced by histological examination showing no lymphocytes or inflammatory cells in any specimen. The histology specimens were harvested at the fusion site, which did not reflect the systemic reaction of these animals. The peripheral blood of these animals should be sampled after implantation of transduced BMDMSCs to detect the inflammatory cytokines, which would more effectively reflect the systemic effects of the implanted vectors on the animals. 

## 4. Materials and Methods

This animal study was approved by the Institutional Animal Care and Use Committee of Change Gung Memorial Hospital. (Approval number: 2012121912; valid period: 20130801 to 20150731).

### 4.1. Isolation of Rabbit Bone Marrow Cells and Culture of Rabbit Bone Marrow Stem Cells

Bone marrow cells were harvested from the iliac crest of New Zealand White Rabbits for allogenic transplantation. The rabbits were anesthetized by intramuscular injection of Zoletil (Virbac Laboratories, Carros, France) at a dose of 10 mg/kg, and 10 mL of bone marrow was aspirated. The bone marrow was flushed with Dulbecco’s Modified Eagle Medium (DMEM). The cell suspension was centrifuged to obtain cells. The cell layer was cultured in DMEM, supplemented with glutamine and 10% heat-inactivated fetal bovine serum (FBS). The cells were plated into a 10-mm culture dish and incubated in 5% CO_2_ at 37 °C. The culture medium was changed twice a week along with removal of non-adherent cells. When the confluence reached 80%–90%, the adherent cells were subcultured at a split ratio of 1:3. Cells of passage 3 were used for transduction with baculoviral vectors.

### 4.2. Construction and Production of Baculoviral Vectors

The recombinant baculovirus expressing BMP-7 under the cytomegalovirus immediate early (CMV-IE) promoter was established and designed as Bac-BMP-7. Concisely, the CMV-IE promoter was produced from pcDNA3.1 (Invitrogen, Carlsbad, CA, USA) by a polymerase chain reaction (PCR)-amplified and subcloned into the pFastBac DUAL vector (Invitrogen). The human *BMP-7* gene was amplified and cloned downstream of the CMV-IE promoter. According to the manufacturer’s instructions, the plasmid keeping the BMP-7 gene was employed to establish the recombinant baculoviruses using the Bac-To-Bac system (Invitrogen). The virus was further spread in *Spodoptera frugiperda* (Sf-9) cells grown in suspension (200 mL) with TNM-FH medium supplemented with 10% FBS. After transfection, the virus particles were collected on day 3 and were concentrated at 80,000× *g* for 2 h at 4 °C and filtered through a 0.22-μm pore filter. The viral titer was determined using the end-point dilution method.

### 4.3. Transduction of Bone Marrow Derived Mesenchymal Stem Cells (MSCs) with Baculoviral Vectors

The bone marrow MSCs were seeded onto 100-mm culture dishes at a density of 1 × 10^6^ cells and were cultured overnight. The culture medium was removed, and the cells were washed once with PBS before transduction. A certain volume of virus suspension at the multiple of infection (MOI) 25 was mixed with DMEM to adjust the final volume to 3 mL per culture dish. Transduction was launched by directly adding the virus mixture to the cells, followed by gentle shaking on a rocking plate for 4 h at 25 °C–27 °C. After 24 h incubation, the virus suspension was removed; the cells were washed with DMEM and incubated at 37 °C.

### 4.4. Quantification of in Vitro BMP-7 Production by Enzyme-Linked Immunosorbent Assay (ELISA)

In vitro BMP-7 production by BMDMSCs transduced with baculo-BMP-7 vectors was quantified in the culture medium using an ELISA kit (R & D Systems, Minneapolis, MN, USA), according to the manufacturer’s instruction. At one day, three days, five days, and seven days after transduction, the culture media from BMDMSCs transduced with the Baculo-BMP-7 vector, BMDMSCs transduced with a baculoviral vector without the BMP sequence, and untransduced BMDMSCs were collected to analyze BMP-7 production. This test was performed to determine whether Baculo-BMP-7-BMDMSCs could overexpress BMP-7.

### 4.5. Implantation of Test Composite into the Rabbit Lumbar Posterolateral Fusion Site

#### 4.5.1. Treatment Groups and Surgery Procedure

Group I (collagen-β-TCP-HA) (*n* = 6): The rabbits were implanted at each L4-5 posterolateral area with a collagen-β-TCP-HA carrier.

Group II (collagen-β-TCP-HA/BMDMSCs) (*n* = 6): The rabbits were implanted at each L4-5 posterolateral area with a collagen-β-TCP-HA carrier containing 2 × 10^7^ BMDMSCs.

Group III (collagen-β-TCP-HA/Baculo-BMP-7-BMDMSCs) (*n* = 6): The rabbits were implanted at each L4–5 posterolateral area with a collagen-β-TCP-HA carrier containing 2 × 10^7^ Baculo-BMP-7-BMDMSCs.

The carrier used in this study for mesenchymal stem cell delivery was a collagen-mineral composite containing type-1 collagen, β-tricalcium phosphate, and hydroxyapatite (FormaGraft, NuVasive Inc., San Diego, CA, USA). The collagen-β-TCP-HA carrier was sterilized with 70% alcohol and pre-wetted overnight with fresh medium. Before the surgery, the cells for implantation were washed, trypsinized, centrifuged, and counted. A total of 2 × 10^7^ cells for each segment were concentrated into a volume of 500 μL and placed into the collagen-β-TCP-HA carrier for the in vivo study. These test cells were successfully seeded on the scaffold, which was confirmed by SEM, and were used for further animal studies.

The rabbits were anesthetized with an intra-muscular injection of Zoletil (10 mg/kg) (Virbac Laboratories). The rabbits were placed in a prone position, and their back was shaved and disinfected with povidone-iodine. A posterior midline incision was made in the skin. Two separate incisions in the lumbar fascia were made at 2 cm from the midline and deepened into the L5–6 transverse process. The transverse processes were decorticated with a high-speed burr. The test materials were then implanted in the inter-transverse process space on each side. The fascia and the skin were closed with 3-0 Vicryl sutures. A bacitracin-neomycin ointment was spread on the wound. Post-operatively, the animals received 200 mg cefamezine per day for 3 days. No postoperative bracing was applied. All the rabbits were allowed unlimited activity.

After 2 weeks, all the animals were sacrificed for final evaluation.

#### 4.5.2. Radiographic Analysis

All animals were anesthetized and lumbar antero-posterior radiographs were taken at 4, 8, and 12 weeks. Each of these radiographs was taken under the same conditions (50 kv/160 mA/120 cm/10 mAs). After 12 weeks, all the rabbits underwent 2-mm thin-cut CT scans of the lumbosacral area. Three-dimensional models were created to visualize the fusion mass amid the inter-transverse process. The CT results were used to determine whether the inter-transverse area was fused or not. Two independent observers who were not involved in the index operation performed the radiographic assessment.

#### 4.5.3. Manual Palpation

After radiographic evaluation, all the rabbits were sedated and sacrificed. The lumbar and upper sacral spines were then harvested. The L4-5 segment was palpated and twisted. A gross union was identified when there was no motion across the surgical segment.

### 4.6. Histological Analysis

Histological studies were performed for specimens from each group to evaluate the fusion status between the inter-transverse process areas. The specimens were fixed in formalin for days and were decalcified with a 10% decalcifying HCL solution (Cal-Ex; Fischer Scientific, Fairlawn, NJ, USA) for 7 days. The specimens were then embedded in paraffin and were sectioned at 5 µm in the sagittal plane of the fusion mass. The resulting sections were stained with hematoxylin and eosin or with Masson’s trichrome. The components of the fusion mass and the interface between the original transverse process and the fusion mass were assessed using standard light microscopy. Any adverse host responses to BMDMSCs or the scaffold were monitored by assessing the infiltration of lymphocytes or other inflammatory cells.

### 4.7. Statistical Analyses

The numerical data was compared using the *t*-test. The fusion rate was compared by the χ-square test. Differences were considered significant at *p*-values of less than 0.05.

## 5. Conclusions

In summary, this study demonstrated BMDMSCs could be genetically modified with the baculoviral vector containing *BMP-7* gene and functionally secreted BMP-7 protein in vitro. The collagen-β-TCP-HA scaffold adhered with these BMDMSCs containing the *BMP-7* gene achieved spinal posterolateral fusion in the rabbit model.
